# Prognostic value of the liver fibrosis marker fibrosis-5 index in patients with severe isolated tricuspid regurgitation: comparison with fibrosis-4 index

**DOI:** 10.1007/s00380-023-02268-3

**Published:** 2023-04-23

**Authors:** Mitsutaka Nakashima, Toru Miyoshi, Machiko Tanakaya, Takaaki Saito, Yusuke Katayama, Satoru Sakuragi, Yoichi Takaya, Hiroshi Ito

**Affiliations:** 1 Department of Cardiovascular Medicine, Okayama University Graduate School of Medicine, Dentistry and Pharmaceutical Sciences, 2-5-1 Shikata-Cho, Kita-Ku, Okayama, 700-8558 Japan; 2 Department of Cardiovascular Medicine, National Hospital Organization Iwakuni Clinical Center, Iwakuni, Japan

**Keywords:** Liver disorder, Fibrosis-4 index, Fibrosis-5 index, Isolated tricuspid regurgitation, Major adverse cardiac events

## Abstract

The fibrosis-4 index (FIB4), a liver fibrosis maker, has been shown to be associated with the prognosis in patients with severe isolated tricuspid regurgitation (TR). Recent study showed that the fibrosis-5 index (FIB5), which was calculated by albumin, alkaline phosphatase, aspartate transaminase, alanine aminotransferase and platelet count, had better prognostic value than FIB4 in patients with heart failure. The aim of this study was to evaluate the usefulness of FIB5 index for predicting prognosis in patients with severe isolated TR and compare the prognostic value between the FIB4 and the FIB5 in those patients. This was a dual-center, retrospective study. 113 consecutive outpatients with severe isolated TR (mean age, 65.8 years; 47.8% male) were analyzed. Major adverse cardiovascular events (MACEs) were defined as the composite of cardiovascular death, hospitalization for heart failure, myocardial infarction, and stroke. During a median follow-up of 3.0 years, 41 MACEs occurred. Patients with MACEs had a lower the FIB5 than patients without MACEs. The multivariate Cox analysis revealed that the FIB5 < -4.30 was significantly associated with higher incidence of MACEs after adjusted by confounding factors. Receiver-operating characteristic curve analyses showed that prognostic values did not differ between the FIB5 and the FIB4 in whole patients and in patients aged ≥ 70 years; while, in patients aged < 70 years, the FIB5 had better prognostic value than the FIB4. The FIB5 may be a useful predictor of MACEs in patients with severe isolated TR.

## Introduction

Isolated tricuspid regurgitation (TR) may be asymptomatic, even if its degree is classified as severe TR [1]. There is still less evidence in patients with isolated TR about the natural history [1–3]. Although significant isolated TR was associated with worse prognosis in both primary and secondary TR, the factors predicting their prognosis were not still established [4]. In patients with severe TR, organ damage caused by right sided heart failure has been reported to be associated with worse prognosis in patients with severe isolated TR [5–8]. Especially, estimation of the liver disorder is important for the management of severe isolated TR [9].

As a surrogate marker of liver fibrosis, the fibrosis-4 index (FIB4), calculated by age, platelet (PLT) count, aspartate aminotransferase (AST) and alanine aminotransferase (ALT), has been widely used in patients with liver diseases [10–12]. Recently, the FIB4 has been also reported to be associated with the prognosis in patients with heart failure [13–16]. In addition, we had reported that the FIB4 was associated with prognosis in patients severe isolated TR [17]. However, because the FIB4 includes age, the FIB4 would be largely influenced by age. Indeed, the accuracy of the FIB4 for the estimation of liver fibrosis is indicated to be reduced among elderly patients with liver disease [18]. Recently, the fibrosis-5 index (FIB5), which was calculated by PLT count, AST, ALT, albumin and alkaline phosphatase (ALP), has been reported as a better scoring index for liver fibrosis [19].

Recently, the FIB5 was shown to be associated with the prognosis in patients with heart failure [20]. However, the association between the FIB5 and the prognosis for patients with severe isolated TR has not been elucidated. The aim of this study was to evaluate the usefulness of FIB5 index for predicting prognosis in patients with severe isolated TR and compare the prognostic value between the FIB4 and the FIB5 in those patients.

## Materials and methods

### Study patients

**Fig. 1:**
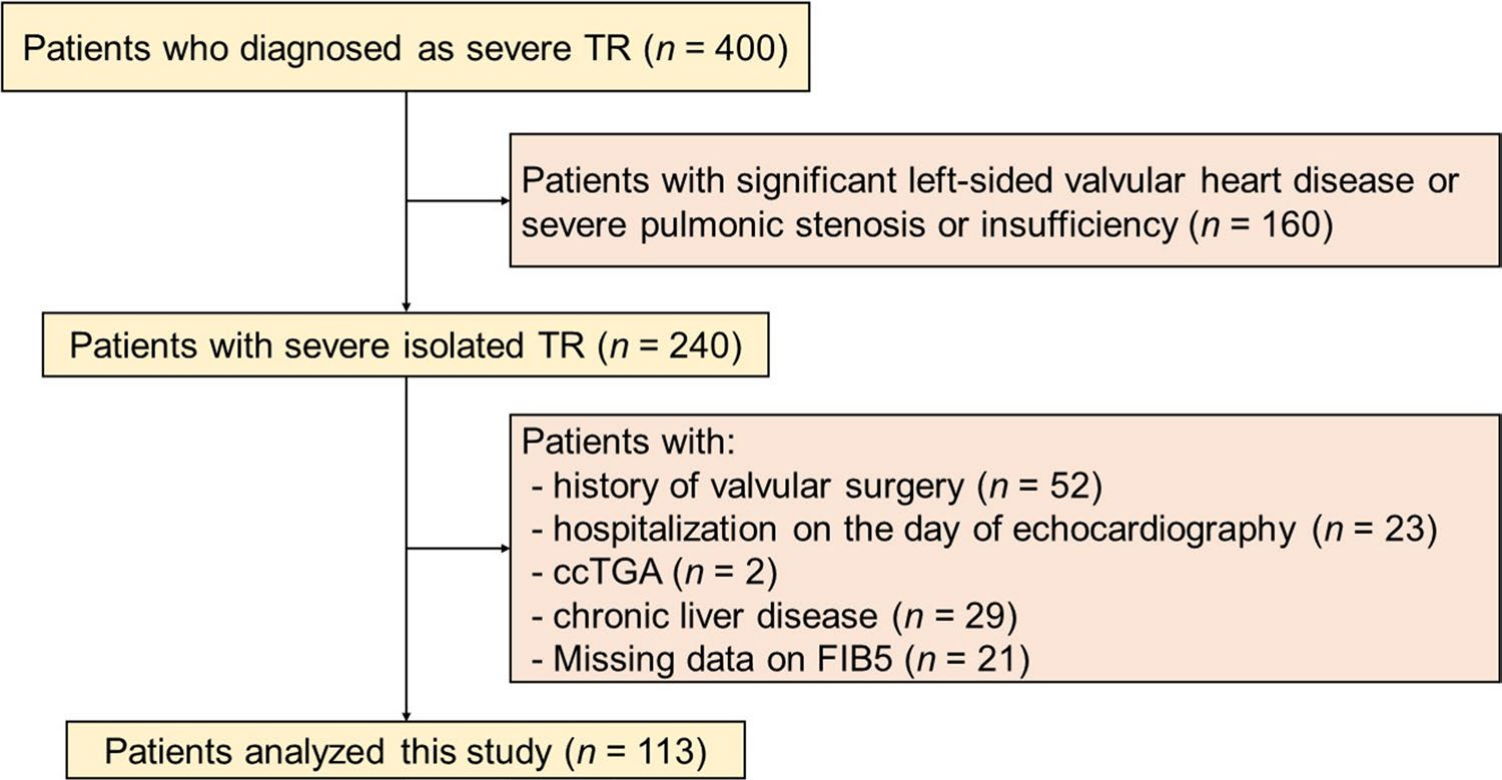
Flow diagram of patient selection. Among 400 outpatients with severe TR, we identified 240 patients with isolated TR. From these, those with history of valvular surgery, decompensated HF that required hospitalization on the day of echocardiography, ccTGA, chronic liver disease and missing data on FIB5 were excluded. A total of 113 patients were finally analyzed. *TR* tricuspid regurgitation; *ccTGA* congenitally corrected transposition of great arteries; *FIB5* fibrosis-5 index

This was a dual-center, retrospective study. 400 outpatients with severe TR were diagnosed at the Iwakuni Clinical Center from January 2011 to December 2019 and at the Okayama University Hospital from January 2008 to December 2020. The definition of isolated TR was based on our previous study [17]. Isolated TR was defined as TR without moderate or severe left-side valvular stenosis or insufficiency, or severe pulmonic stenosis or insufficiency [2]. We identified 240 outpatients as severe isolated TR. We excluded patients with a history of valvular surgery (*n = *52), decompensated HF that required hospitalization on the same day of echocardiography (*n = *23), congenitally corrected transposition of great arteries because their tricuspid valves play role of systemic atrioventricular valves (*n = *2) and chronic liver disease (*n = *29). Chronic liver diseases were defined as the presence of pre-existing liver disease and/or history of treatment based on the blood examination results and medical records reviewed by a hepatologist. Patients with missing data on FIB5 index were also excluded (*n = *21). Finally, 113 outpatients with isolated severe TR were analyzed. Figure 1 shows the flow diagram of the study design.

This investigation conformed to the principles outlined in the Declaration of Helsinki and was approved by the institutional review boards of Iwakuni Clinical Center (0264) and Okayama University Graduate School of Medicine (2206–020). The requirement for informed consent was waived because of the low-risk nature of the study and because consent could not be directly obtained from all enrolled patients.

### Echocardiography and laboratory data

Echocardiography was performed by experienced technicians using an iE33 (Philips Japan, Ltd., Tokyo, Japan) and Aplio echo machine (Canon Medical Systems, Otawara, Japan). The technicians who performed echocardiography were blinded to the FIB5 index and the FIB4 index. The measurement of echocardiographic parameters was based on the American Society of Echocardiography guideline [21]. The severity of TR was divided into three grades of “mild”, “moderate” and “severe” based on the American Society of Echocardiography guideline, as same with our previous study [17, 22]. Left atrial volume index, left ventricular end-diastolic diameter, left ventricular end-systolic diameter, tricuspid regurgitation pressure gradient and diameter of inferior vena cava were measured using general methods. We measured left ventricular ejection fraction (LVEF) using the modified Simpson technique with B-mode presentation in the apical two-chamber and four-chamber views. Peak early diastolic velocities (*E*) of LV inflow and early diastolic myocardial velocities (*e*ʹ`) were also measured, and the ratio of *E* to *e*ʹ (*E*/*e*ʹ) was calculated. From the apical approach, we measured the tricuspid annular plane systolic excursion (TAPSE) and tricuspid lateral annular systolic velocity to assess RV function.

The blood examinations on the same day of echocardiography were evaluated. The routine laboratory blood examinations and measurements of N-terminal pro-brain natriuretic peptide (NT-pro-BNP) or brain natriuretic peptide (BNP) were performed using an automated analyzer at Iwakuni Clinical Centre and Okayama University Hospital. Estimated glomerular filtration rates (eGFR) were calculated as follows: 194 × creatinine − 1.094 × age − 0.287 for men and 194 × creatinine − 1.094 × age − 0.287 × 0.739 for women.

### Calculation of the FIB5 and the FIB4

The FIB5 index was calculated as follows: [albumin (g/L) × 0.3 + PLT count (10^9^/L) × 0.05] − [ALP (U/L) × 0.014 + AST to ALT ratio × 6 + 14] [19, 20]. The FIB4 index was also calculated as follows: age (years) × AST (U/L)/[ALT (U/L)^1/2^ × PLT count (10^9^/L)] [10]. Both the FIB5 index and the FIB4 index were calculated using the blood examinations on the same day of echocardiography. The lower FIB5 index scores and the higher FIB4 index scores indicate worse values of liver conditions.

### Clinical outcomes

The incidence of major adverse cardiovascular events (MACEs) was investigated by retrospective review of the medical records or telephone interviews when laboratory data were blinded. Off 113 patients who were enrolled in this study, 76 patients (67.3%) were investigated by retrospective review of the medical records and 37 patients (32.7%) were investigated by telephone interviews. MACEs were defined as a composite of the following clinical outcomes: cardiac death; hospitalization for HF; non-fatal non-ST elevation/ST elevation myocardial infarction; ischemic/non-ischemic stroke; heart or lung transplantation. Cardiac death was defined as death caused by any of the following: acute coronary syndrome; heart failure; arrhythmic death; and unclear causes of death for which a cardiac origin could not be excluded [17].

### Statistical analysis

Categorical variables are presented as numbers (%) and were compared using the *χ*^2^-test or Fisher’s exact test as appropriate. Continuous variables that were normally distributed are presented as means ± standard deviation and were compared using Student’s t-test. Continuous variables that were not normally distributed are presented as medians with interquartile ranges (IQRs) and were compared using the Mann–Whitney *U* test. Data normality was evaluated using the Shapiro–Wilk test. To assess MACEs, a receiver-operating characteristic (ROC) curve analysis was performed for the FIB5 and the FIB4. The optimal cutoff value was defined as the point maximizing the Youden index (= max [sensitivity + specificity − 1]). We classified patients into two groups according to the optimal cutoff values of the FIB5 index. Cumulative event-free rates of the clinical endpoints during follow-up were compared between groups using Kaplan–Meier curves, post hoc comparisons, and log-rank tests. The effects of the FIB5 as a categorized variable divided by above the optimal cutoff value on MACEs were evaluated using the Cox proportional hazard analysis. The results are reported as hazard ratios (HRs) and 95% confidence intervals (CIs). The multivariate Cox proportional hazard analyses were performed adjusted variables which were considered as confounding factors for MACEs by three models as follows. Model 1 was adjusted by patient general characteristics; age and gender. Model 2 was adjusted by laboratory findings; hemoglobin and eGFR. Model 3 was adjusted by left ventricular and right ventricular function assessed by echocardiography: LVEF, E/e’ and TAPSE. Since the FIB4 includes age, further analyses were performed to evaluate the influence of age on the impact of the FIB5 and the FIB4 for MACEs. We divided patients to groups by median value of age; a group of patients with less than median of age, and the other group of patients with median or more than median of age. The univariate Cox proportional hazard analysis was additionally evaluated for each group, respectively. The effects of the FIB5 index as a categorized variable divided by the optimal cutoff value of each group were evaluated. The area under the ROC curve was compared between the FIB5 and FIB4. We additionally compared the area under the ROC curve each group divided by the median value of age. Statistical significance was set at *p < *0.05. These analyses were performed using SPSS statistical software (version 25; IBM Corp., Armonk, NY, USA) and the R statistical package (version 3.6.3; R Foundation for Statistical Computing, Vienna, Austria).

## Results

### Patient characteristics

Table 1 shows the clinical characteristics of all patients. Study patients included 54 men (47.8%) with a mean age of 65.8 years. The median value of age was 70 years. Primary TR was observed in 23 patients (endocarditis, *n = *1; pacing lead, *n = *9; prolapse, *n = *2; iatrogenic perforation of the tricuspid valve *n = *1; Ebstein anomaly, *n = *10). Secondary TR was observed in 90 patients (left heart disease, *n = *8; right ventricular volume overload, *n = *2; right ventricular cardiomyopathy, *n = *5; pulmonary arterial hypertension, *n = *4; chronic lung disease, *n = *5; pulmonary thromboembolism, *n = *7; left-to-right shunt, *n = *20, atrial fibrillation, *n = *38; compression by pectus excavatum, *n = *1). 67 patients (59.3%) had atrial fibrillation, and 36 patients (31.9%) had history of heart failure. Median LVEF and E/*e*ʹ were 63.0% and 10.3, respectively. Mean TAPSE and tricuspid lateral annular systolic velocity were 18.8 mm and 11.1 cm/s, respectively.

**Table 1:** Baseline clinical characteristics of this study

Variables	*n = *113
Age, years	65.8 ± 18.6
Male	54 (47.8)
Body mass index, kg/m^2^	20.5 ± 2.8
NYHA class, n (%)	
I/II/III/IV	32/42/37/2
Etiology of TR	
Primary	23 (20.4)
Secondary	90 (79.6)
Hypertension	34 (30.1)
Diabetes mellitus	19 (16.8)
Atrial fibrillation	67 (59.3)
Implantation of CIEDs	34 (30.1)
History of heart failure	36 (31.9)
COPD	5 (4.4)
Ischemic heart disease	10 (8.8)
Medications on admission	
β-Blockers	56 (49.6)
ACEIs/ARBs	32 (28.3)
MRA	51 (45.1)
Echocardiographic data	
LAVI, mL/m^2^	49.5 (33.0–66.0)
LVDd, mm	42.9 ± 7.2
LVDs, mm	28.0 (24.0–33.0)
LVEF, %	63.0 (55.0–69.0)
*E*/*e*ʹ	10.3 (8.5–13.2)
TAPSE, mm	18.8 ± 6.6
*S*ʹ, cm/s	11.1 ± 3.8
TRPG, mmHg	32.0 (25.0–44.0)
Maximum IVC diameter, mm	19.0 ± 6.0
IVC respiratory change rate, %	43.7 ± 19.3

Data are presented as the number (%), mean ± standard deviation, or median (25th–75th percentile)

*NYHA* New York Hear Association; *TR* tricuspid regurgitation; *CIEDs* cardiovascular implantable electronic devices; *COPD* chronic obstructive pulmonary disease; *ACEIs* angiotensin-converting enzyme inhibitors; *ARBs* angiotensin II receptor blockers, *MRA* mineralocorticoid receptor antagonist; *LAVI* left atrial volume index; *LVDd* left ventricular end-diastolic diameter; *LVDs* left ventricular end-systolic diameter; *LVEF* left ventricular ejection fraction; *E*/*e*ʹ early diastolic filling velocity/early diastolic velocity of the mitral annulus; *TAPSE* tricuspid annular plane systolic excursion; *S*ʹ, tricuspid lateral annular systolic velocity; *TRPG* tricuspid regurgitation pressure gradient; *IVC* inferior vena cava

### Relationship between the FIB5 and clinical outcomes

During a median follow-up of 3.0 years, 41 MACEs occurred (cardiovascular death, *n = *4; hospitalization for HF, *n = *31; myocardial infarction, *n = *1; stroke, *n = *4; lung transplantation for pulmonary hypertension, *n = *1). Tricuspid valve surgery was performed for seven patients. Two patients underwent tricuspid valve surgery after hospitalization for HF. One patient had cardiovascular death after tricuspid valve surgery. One patient had hospitalization for HF after tricuspid valve surgery. Table 2 shows laboratory data including the FIB5 of the patients between groups with MACEs and without MACEs during follow-up period. PLT count and serum albumin were significantly lower (*p = *0.023 and *p = *0.049, respectively) and ALP was significantly higher (*p = *0.007) in patients with MACEs than in patients without MACEs. Serum creatinine and eGFR also significantly differ between the groups (*p = *0.001 and *p = *0.013, respectively). The FIB5 was significantly lower in patients with MACEs than in patients without MACEs (− 8.39 [− 11.35 to − 5.56] and − 3.54 [− 7.93 to − 1.55], *p = *0.001). The FIB4 was also significantly higher in patients with MACEs than in patients without MACEs (3.47 [1.81–4.42] and 2.07 [1.54–2.92], *p = *0.010). Hemoglobin, AST, ALT and total bilirubin did not differ between the groups. NT-proBNP levels (*n = *32) was 1940 [1378–3166] pg/mL in patients with MACEs and 1578.5 [740–2345] pg/mL in patients without MACEs (*p = *0.193). BNP levels (*n = *72) were 331 [185–508] pg/mL in patients with MACEs and 185 [81–376] pg/mL in patients without MACEs (*p = *0.014).

**Table 2:** Laboratory data and the FIB5 index of this study

Variables	All patients *N = *113	CV events		*p* value
		Present *N = *41	Absent *N = *72	
Hemoglobin, g/dL	12.4 ± 1.9	12.2 ± 2.2	12.6 ± 1.8	0.319
PLT count, 10^9^/L	183.4 ± 67.6	164.3 ± 59.9	194.3 ± 69.6	0.023
AST, IU/L	26 (21–33)	29 (22–36)	25 (20.5–32.5)	0.092
ALT, IU/L	17 (13–24)	17 (13–30)	16.5 (13–22.5)	0.374
ALP, IU/L	258 (196–368)	300 (201–411)	239.5 (180.5–300.5)	0.007
Serum albumin, g/dL	4.1 (3.8–4.4)	4.0 (3.7–4.3)	4.2 (3.9–4.4)	0.049
FIB5	− 5.90 (− 9.91 to − 2.27)	− 8.39 (− 11.35 to − 5.56)	− 3.54 (− 7.93 to− 1.55)	0.001
FIB4	2.38 (1.56–3.77)	3.47 (1.81–4.42)	2.07 (1.54–2.92)	0.010
Total bilirubin, g/dL	0.8 (0.6–1.1)	0.9 (0.6–1.2)	0.8 (0.5–1.1)	0.274
Serum creatinine, mg/dL	0.9 (0.7–1.1)	1.1 (0.9–1.4)	0.9 (0.7–1.0)	0.001
eGFR, mL/min/1.73 m^2^	57.3 ± 21.7	50.4 ± 22.8	61.1 ± 20.2	0.013

Data are presented as mean ± standard deviation, or median (25th–75th percentile)

*CV* cardiovascular; *PLT count* platelet count; *AST* aspartate aminotransferase; *ALT* alanine aminotransferase; *ALP* alkaline phosphatase; *FIB5* fibrosis-5 index; *FIB4* fibrosis-4 index; *eGFR* estimated glomerular filtration rate

**Fig. 2:**
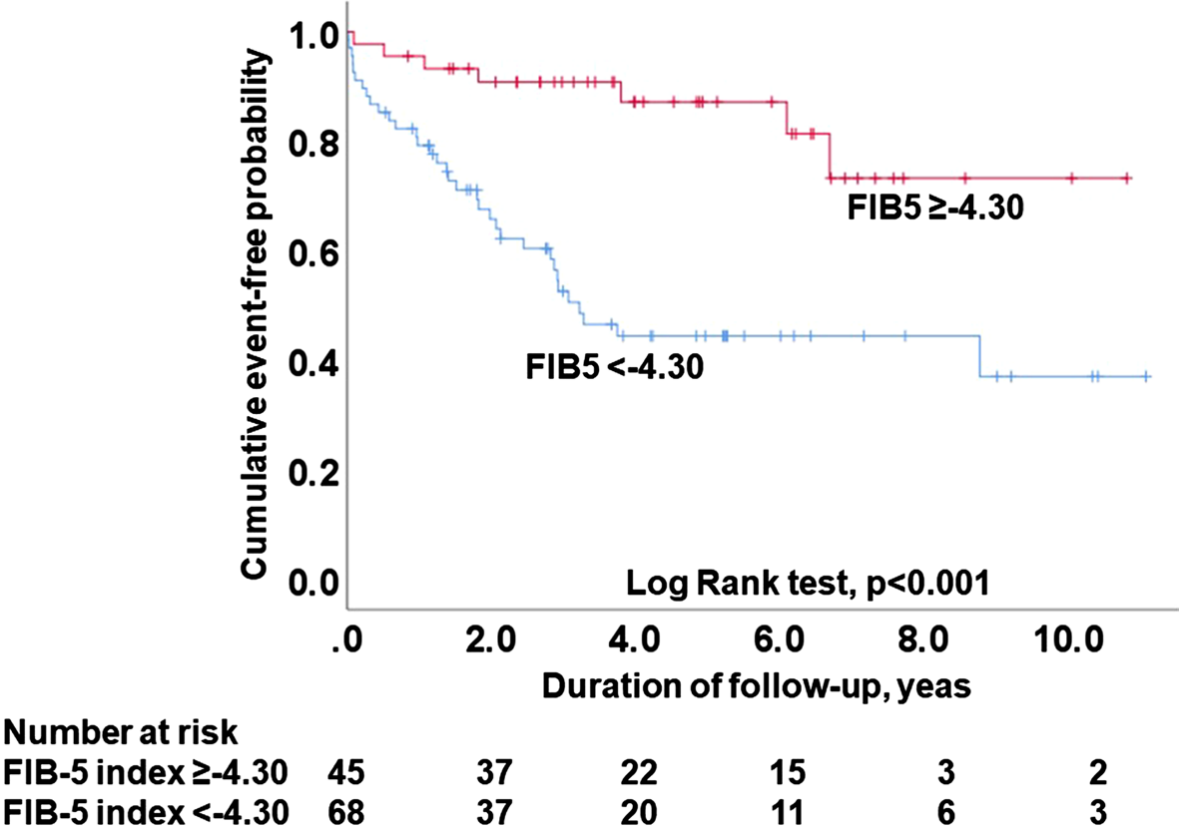
Kaplan–Meier analysis of the event-free rate of major cardiovascular events in the groups divided by the optimal cutoff value of the FIB5. *FIB5* fibrosis-5 index

The ROC curve analysis showed that the C-statistic of the FIB5 for MACEs prediction in whole patients was 0.696 (95% CI 0.596–0.796; *p = *0.001), with a sensitivity of 82.9% and a specificity of 52.8%, and that the value maximizing the Youden index was − 4.30. Figure 2 shows the Kaplan–Meier analyses of patients stratified according to the above optimal cutoff value for the FIB5 index score. Patients with FIB5 < − 4.30 had a significantly poor cardiovascular prognosis compared with patients with higher FIB5 ≥ − 4.30 (*p < *0.001). As shown in Table 3, the Cox regression analysis showed that the MACE incidence was significantly associated with FIB5 as a categorized variable divided by optimal cutoff in both univariate and multivariate analyses adjusted by confounding factors.

**Table 3:** Univariate and multivariate Cox regression analyses of major adverse cardiovascular events

Variable	Hazard ratio	95% Confidence interval	*p* value
FIB5 < − 4.30 (univariate)	4.313	1.906–9.762	< 0.001
FIB5 < − 4.30 (Model 1^a^)	4.161	1.785–9.697	0.001
FIB5 < − 4.30 (Model 2^b^)	3.706	1.605–8.559	0.002
FIB5 < − 4.30 (Model 3^c^)	3.494	1.125–10.855	0.030

*FIB5* fibrosis-5 index; *Hgb* hemoglobin; *eGFR* estimated glomerular filtration rates; *LVEF* left ventricular ejection fraction; *E*/*e*ʹ early diastolic velocity/early diastolic myocardial velocity; *TAPSE* tricuspid annular plane systolic excursion

^a^Adjusted by age and gender

^b^Adjusted by Hgb and eGFR

^c^Adjusted by LVEF, *E*/*e*ʹ and TAPSE

After dividing patients by the median value of age (70 years), MACEs were documented for 17 in patients aged < 70 years and for 24 in patients aged ≥ 70 years. The ROC curve analysis showed that the C-statistic of the FIB5 for MACEs prediction in patients aged < 70 years was 0.694 (95% CI 0.536–0.852; *p = *0.024), with a sensitivity of 88.2% and a specificity of 51.4%, and that the value maximizing the Youden index was − 2.27. The ROC curve analysis showed that the C-statistic of the FIB5 for MACEs prediction in patients aged ≥ 70 years was 0.702 (95% CI 0.536–0.852; *p = *0.008), with a sensitivity of 66.7% and a specificity of 78.4%, and that the value maximizing the Youden index was − 8.39. The Cox regression analysis showed that the MACE incidence was significantly associated with FIB5 as a categorized variable divided by above each optimal cutoff both in patients aged < 70 years (HR 4.218; 95% CI 1.860–9.566; *p = *0.001) and in patients aged ≥ 70 years (HR 3.362; 95% CI 1.434–7.881; *p = *0.005).

### Comparison of impact on clinical outcomes between FIB5 and FIB4

**Fig. 3:**
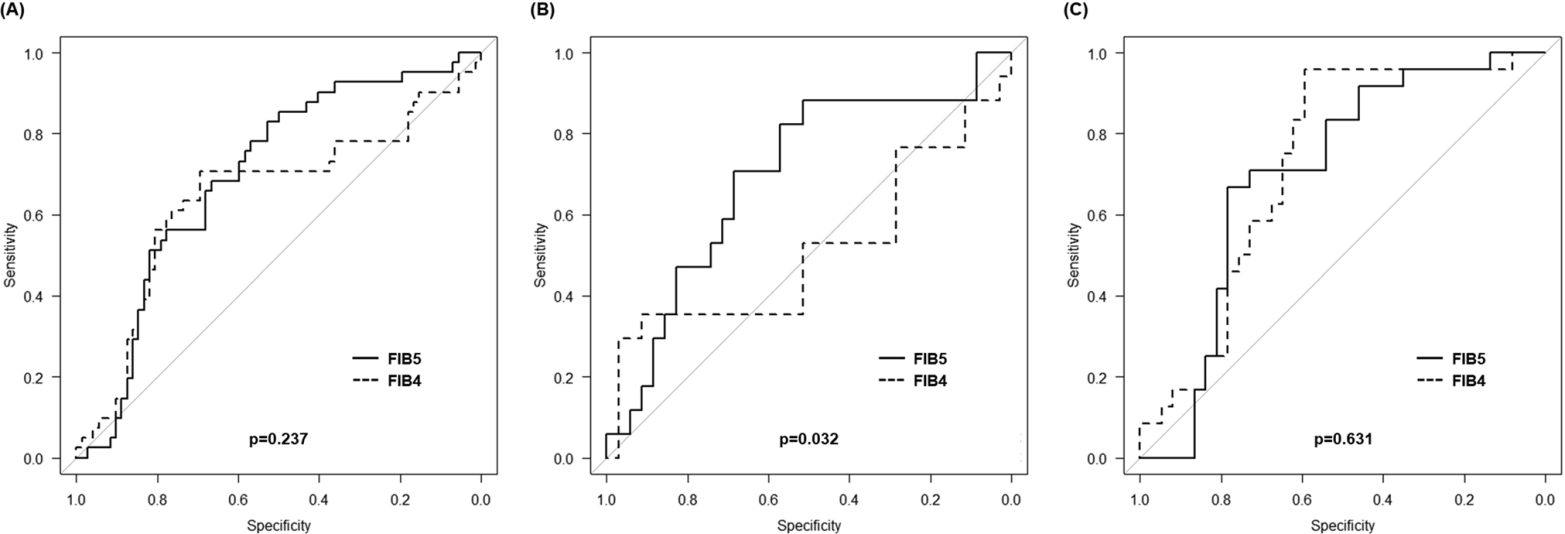
Comparison of the area under the receiver-operating characteristic curve for prediction of MACEs between the FIB5 and the FIB4. **A** The receiver-operating characteristic curve in whole patients, **B** in patients with less than median of age, **C** in patients with median or more than median of age. The predictive value of FIB5 was significantly higher than that of FIB4 in patients with less than median of age. *MACEs* major adverse cardiovascular events; *FIB5* fibrosis-5 index; *FIB4* fibrosis-4 index

The C-statistic of the FIB4 for MACEs prediction in whole patients was 0.645 (95% CI 0.531–0.760; *p = *0.010). Figure 3A shows the ROC curves of the FIB5 and FIB4 for MACEs in whole patients. There was no significant difference in the C-statistics between FIB5 and FIB4 (0.696 and 0.645, *p = *0.237). After dividing patients by the median value of age (70 years), the C-statistics of the FIB4 for MACEs prediction in patients aged < 70 years was 0.513 (95% CI 0.320–0.705; *p = *0.884). The C-statistic of the FIB4 for MACEs prediction in patients aged ≥ 70 years was 0.725 (95% CI 0.595–0.856; *p = *0.003). Figure 3B shows the ROC curves in patients aged < 70 years and Fig. 3C shows those in patients aged ≥ 70 years. The C-statistics of FIB5 were significantly greater than that of the FIB4 in patients aged < 70 years (0.694 and 0.513, *p = *0.032), but not in patients aged ≥ 70 years (0.702 and 0.725, *p = *0.631).

## Discussion

This study investigated the impact of the FIB5 on MACEs in patients with severe isolated TR. A lower FIB5 was significantly associated with a higher incidence of MACE. There were no significant differences in C-statistics between the FIB5 and the FIB4 in patients with whole patients and in patients aged ≥ 70 years. In patients aged < 70 years, the C-statistics of FIB5 was significantly greater than that the FIB4. To the best of our knowledge, this is the first study to evaluate whether the low FIB5 reflects a poor cardiovascular prognosis in patients with severe isolated TR, comparing the FIB4 according to patient’s age.

### FIB5 and MACE incidence in severe isolated TR

The FIB5 index has been developed in order to assess the liver fibrosis in patients with liver diseases [19]. The FIB5 index was also reported to be associated with prognosis in patients with heart failure [20]. The FIB5 differs from the FIB4 index in that the FIB5 includes albumin and ALP and does not include age in the calculation formula. Since both albumin and ALP are also reported to be associated with the prognosis in patients with heart failure respectively, adding these two parameters in calculation formula seems to be helpful for prediction for prognosis in patients with heart failure [23, 24]. The FIB5 does not include age whereas the FIB4 includes, which might lead the usefulness of the FIB5 more versatile than the FIB4. Although age is one of the major risk factors for worse prognosis in patients with heart failure, the utility as a prediction marker of the FIB4 might be limited if the target population includes wide spectrum of pathology and wide range of age [18, 25, 26]. In this study, patients with severe isolated TR were enrolled. The etiologies in this study population included wide spectrum from TR caused by congenital heart disease or pulmonary arterial hypertension [27] which could develop in even young patients to TR caused by left heart disease or atrial fibrillation which could develop in elderly patients. Thus, there was an apprehension that the utility of the FIB4 have a limitation in this study population, although we reported that the FIB4 was significantly associated with the prognosis in patients with severe isolated TR [17]. The FIB5 was also significantly associated with MACEs in patients with severe isolated TR in this study. Even after adjusted by age in a multivariate Cox analysis or after divided by median value of age, the FIB5 showed significant association with MACEs. These findings indicate that the FIB5 could be a feasible prognostic surrogate marker for MACEs in patients with severe isolated TR, regardless patient age.

### Comparison the prognostic values between the FIB5 and the FIB4

In this study, both the FIB5 and the FIB4 were significantly associated with the MACEs in whole patients, as same the previous studies [13–17, 20]. The prognostic values were not significantly different between the FIB5 and the FIB4 in whole study population and in patients aged ≥ 70 years (median value of age in this study). However, in patients aged < 70 years, despite the FIB5 was also significantly associated with MACEs, the FIB4 was not. The prognostic value of the FIB5 significantly outperformed that of the FIB4 in patients aged < 70 years. From these findings, it was indicated that the prognostic impact of the FIB4 might be deceased in younger population whereas that of the FIB5 might be preserved regardless age of patient population. In liver disease, the cutoff value of the FIB4 was suggested to be changed as patient age. The optimal cutoff values of the FIB5 and the FIB4 in patients with cardiovascular disease including isolated TR were still not established. In young patients, the value of the FIB4 will tend to be relatively low score even if their liver dysfunction is high grade. Whether their result could apply to younger patient population is still unknown because the study populations in previous studies which evaluated the prognostic value of the FIB4 in patients with cardiovascular diseases mainly included elderly patients [13–17, 20]. At this point, the FIB5 might be efficient to apply versatilely because its calculation formula does not include age. The further studies are required because the limited sample size in this study could not decide the cutoff value of age that the prognostic value of the FIB4 would be inferior that of the FIB5.

This study sought to evaluate the direct prognostic impact of liver conditions due to right sided heart failure in patients with severe isolated TR. As a result, 287 patients (71.8%) of 400 severe TR patients were excluded from this study. Clinical use of the FIB5 needs to be cautious, because this study included only patients with severe isolated TR. The optimal cutoff and clinical usefulness on the FIB5 in all patients with severe TR should be investigated further.

This study had several limitations. First, the study had a relatively small sample size. Second, this was a retrospective study and there were 21 patients (8.8%) excluded from 240 patients with severe isolated TR because of missing data on FIB5. This might lead to selection bias. Third, we classified TR as only three grades according to the standard American Society of Echocardiography grading scheme, whereas recent classification is suggested to classify the five grades to as mild, moderate, severe, massive, and torrential [22, 28]. Fourth, chronic liver diseases were not completely ruled out because we did not perform additional examinations such as liver biopsy or magnetic resonance imaging for evaluation routinely. It was still unclear whether the FIB5 directly reflects the liver conditions in patients with severe isolated TR as same in patients with liver diseases. Finally, this study included only patients with severe TR. Therefore, our findings could not apply directory to all severe TR patients.

In conclusion, the FIB5 is associated with a high risk of MACEs in patients with severe isolated TR. The prognostic value of the FIB5 significantly outperformed that of the FIB4 in patients with aged < 70 years. In the clinical setting, the FIB5 may be useful for assessing the risk of future cardiovascular events in patients with severe isolated TR, regardless their age.

## Data Availability

The datasets used and analyzed during the current study are not publicly available but are available from the corresponding author on reasonable request.
